# Effective Cell Transfection in An Ultrasonically Levitated Droplet for Sustainable Technology

**DOI:** 10.1002/advs.202203576

**Published:** 2022-08-26

**Authors:** Takahiro Arai, Toshinori Sato, Teruhiko Matsubara

**Affiliations:** ^1^ Department of Biosciences and Informatics Faculty of Science and Technology Keio University 3‐14‐1 Hiyoshi, Kohoku‐ku Yokohama Kanagawa 223–8522 Japan

**Keywords:** acoustic levitation, cell transfection, containerless processing, plastic free, standing wave, trapping, ultrasonics

## Abstract

The levitation methodology, which enables us to operate a contactless reaction without a container, is likely to be a revolutionary technology in the fields of chemistry and biology to reduce the plastic waste in life science laboratories. Here, the authors show that plasmid DNA can be effectively transfected into animal cells in a floating droplet of culture medium levitated using ultrasonic standing waves. The data indicate that there is no significant damage to the plasmid and cells during the levitating transfection time, and the transgene expression efficiency and cellular uptake in the droplet are significantly higher than those in the conventional tube, with and without shaking. These results suggest the consolidation of the endocytic uptake pathway into macropinocytosis, indicating that ultrasonic levitation induced a change in cell characteristics. This study suggests that transfection methodology using ultrasonic levitation has the potential to advance the current experimental procedures in the field of cell engineering, in addition to presenting a revolutionary containerless reactor for sustainable technology.

## Graphical abstract

Transfection methodology using ultrasonic levitation has the potential to alter the current experimental process in the area of cell engineering. Herein, it is reported that plasmid DNA can be effectively transfected into animal cells in floating droplets of culture medium levitated using ultrasonic standing waves by consolidation of the endocytic uptake pathway into macropinocytosis.

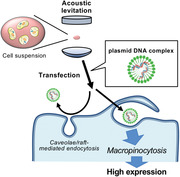

## 1 Introduction

Polymeric materials including polystyrene and polypropylene are commonly used for chemical and biological reactions in laboratories. Because pipette tips, tubes, and dishes are disposable, the plastic waste in laboratories is estimated to be approximately several tens of kg per person in a year.^[^ ^1^ ^]^ For increased sustainability, some scientists are starting to change their habits and to eliminating single‐use plastics through reduction or reuse in laboratories.^[^ ^2^ ^]^ Acoustic levitation is a powerful technology for the contactless manipulation of objects with sizes of a few millimeters in both the gas and liquid phases, regardless of the type of material, including rigid spheres and liquid droplets.^[^ ^3^, ^4^ ^]^ Contactless handling of objects using levitation technology has the potential to be advantageous for treating chemical compounds, delicate biological samples, and microorganisms without container, aiding in the reduction of the plastic waste in laboratories. Droplet‐based microfluidics with acoustic levitation has been applied for the study of liquid marble coalescence,^[^ ^5^ ^]^ fabrication of the nanoparticle self‐assembly using an air−water interface,^[^ ^6^ ^]^ and bottom‐up creation of artificial cells.^[^ ^7^ ^]^ In the field of microreactions, the use of levitated droplets for chemical reactions, such as click chemistry,^[^ ^8^ ^]^ alkaline phosphatase hydrolysis,^[^ ^9^ ^]^ peroxidase‐catalyzed colorimetric reactions,^[^ ^8^ ^]^ and restriction enzyme digestion^[^ ^8^ ^]^ has been reported. Additionally, this technology has been applied in analytical studies of biological samples including Raman spectroscopy of red blood cells and malaria‐infected cells,^[^ ^10^ ^]^ atmospheric pressure chemical ionization mass spectrometry,^[^ ^11^ ^]^ and structural analysis of apoferritin using synchrotron small‐angle X‐ray scattering.^[^ ^12^ ^]^ Animal cell handling using acoustic levitation in the liquid phase is now being applied in regenerative medicine. Nakao et al. applied acoustic levitation to form scaffold‐free three‐dimensional (3D) cell aggregates using C2C12 cells in a culture medium.^[^ ^13^ ^]^ Cheng et al. arranged PC12 cells with 3D micropatterns using a cross‐linked gelatin hydrogel.^[^ ^14^ ^]^ These reports indicate that cell handling using acoustic levitation has a great potential in a variety of biological applications, such as cell accumulation, chemical reactions, and analytical chemistry.

The use of acoustic levitation for animal cell handling in the air (gas phase) has received increased attention, and some attempts have been made to use floating droplets for cell handling. Santesson et al. levitated small droplets (0.5 µL) containing 3–15 individual cells acoustically, and the cell response was detected by the changes in pH within a few minutes.^[^ ^15^, ^16^ ^]^ Foresti et al. developed a system for continuous planar transport and processing of multiple droplets and performed a contactless DNA transfection into cells by droplet coalescence and mixing.^[^ ^17^, ^18^ ^]^ However, the cells treated by levitation were interacted with DNA within several minutes, and their transfection efficiency was lower than that of conventional procedures. In the present study, we investigated cell transfection conditions using a floating droplet levitated with ultrasonic waves and demonstrated its transfection efficacy. We evaluated the transgene expression efficiency using a luciferase and an enhanced green fluorescent protein (EGFP) genes, and investigated the cellular uptake pathway during the transfection of the droplet. Cell transfection is an essential technique in the field of life sciences; therefore, an effective contactless transfection method can potentially advance this field.

## 2 Results

### 2.1 The Design of the Cell Transfection System in A Floating Droplet

**Figure 1:**
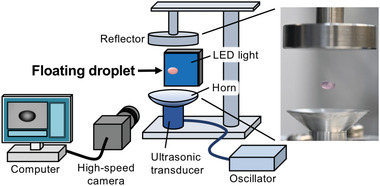
Illustration of the ultrasonic levitation instruments. A standing wave was generated between the transducer and reflector, and the levitated droplet was observed using a high‐speed camera to calculate its volume. Right, a snapshot of an ultrasonically levitated droplet (purple) with a cell and pDNA complex suspension.

**Figure 2:**
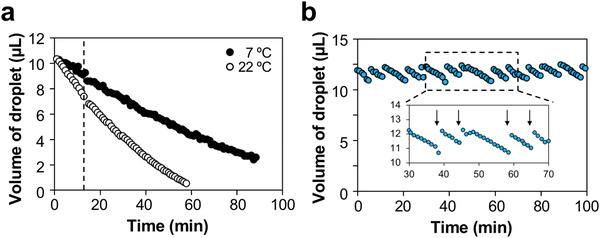
Changes in the volume of the water droplet over time at 7 and 22 °C a) and at 22 °C with the addition of sterile water every 5–10 min b). The humidity of the air around the droplets was not controlled. Broken line, 15 min. Arrow, addition of water.

A single‐axis ultrasonic levitator with a frequency of 60 kHz was combined with a high‐speed camera, and its volume was monitored in real‐time by shooting the floating droplet (**Figure** **1**). Levitating a water droplet for more than 20 min at 25°C resulted in obvious water evaporation in the droplet. Ten microliters of water were initially stably levitated at 22°C and 7°C using our system; however, we observed water evaporation (**Figure** **2**a). The reduction speed of the droplet volume was dependent on the temperature. At 22°C and 7°C, 3.6 µL and 1.8 µL of water were evaporated during the first 15 min of the procedure, respectively. After 60–90 min, the volume of these droplets was less than 0.5 µL. These results indicated that replenishment of evaporated water was required regularly to maintain the volume of the droplet during transfection. Because conventional transfection time is 4 h, the sterile water was added every 5–10 min to maintain the volume (approximately 10 µL) of the droplet (Figure 2b). When the water was added to a droplet using a micropipette, the droplet is temporarily swung slightly from side to side and remained at rest again within several seconds. In addition, the temperature of the droplet was consistent with that of the surrounding environment (22°C and 7°C) during 4 h of levitation.

### 2.2 The Floating Droplet does not Damage the pDNA and Cells

**Figure 3:**
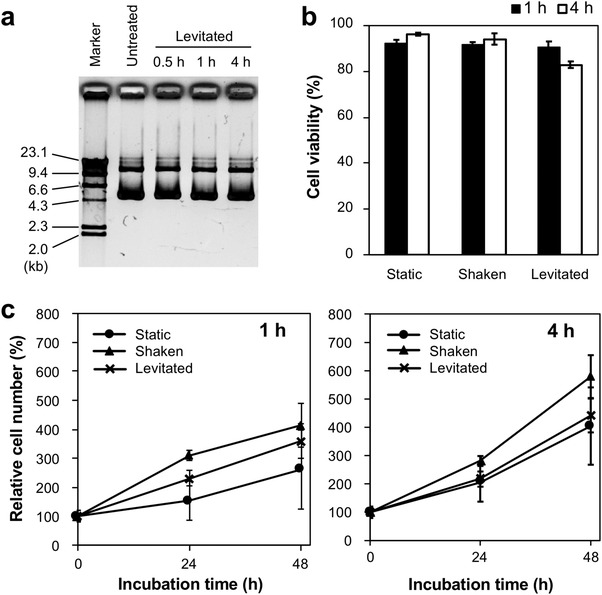
Influence of ultrasonic levitation on the stability of pDNA and cell viability. a) Agarose gel electrophoresis of pDNA exposed to transfection conditions. The pDNA solution was ultrasonically levitated and electrophoresed on a 1.0% agarose gel in Tris‐acetate‐EDTA (TAE) buffer at 100 V for 30 min. Marker: *λ*/HindIII digest. b) Trypan blue exclusion assay. Huh‐7 cells were ultrasonically levitated for 1 or 4 h and then mixed with an equal amount of 0.4% trypan blue solution in PBS. Blue (dead) and white (living) cells were counted using a hemocytometer. Mean ± standard deviation (*n* = 6). c) Cell proliferation assay. Huh‐7 cells were ultrasonically levitated for 1 h or 4 h and then seeded into a 96‐well plate at a density of 3000 cells per well. After 24–48 h of incubation, cell viability was measured. Data are presented as mean ± standard deviation (*n* = 6).

Ultrasonic cavitation damages DNA and cells, and the intensity of the damage is directly related to the ultrasonic power and frequency.^[^ ^19^, ^20^ ^]^ To confirm that plasmid DNA (pDNA) was not damaged during the transfection period in the levitation system, the stability of pDNA in a floating droplet was evaluated by agarose gel electrophoresis. Even after 4 h of levitation, no additional bands were observed, indicating that significant pDNA degradation was not observed (**Figure** **3**a). Miller reported that there was no damage to DNA in cultured Chinese hamster ovary cells with an ultrasonic exposure at <2 W cm^−2^ spatial peak temporal average intensity.^[^ ^19^, ^21^ ^]^ The ultrasonic exposure in the present study was performed at an intensity of less than 1.6 W cm^−2^; hence, it is reasonable that there was no pDNA degradation.^[^ ^8^ ^]^ In addition, because the acoustic impedance of water (1.48 × 10^6^ kg m^−2^ s^−1^) is substantially higher than that of air (430 kg m^−2^ s^−1^), most of the sound energy may not reach the inner side of the droplet due to reflection at the air–water interface of the droplet.^[^ ^22^ ^]^

The viability of human hepatocellular carcinoma (Huh)‐7 cells in a floating droplet was evaluated using the trypan blue exclusion assay and a tetrasodium‐based cell proliferation assay. Because of the internal flow observed in the levitated droplet,^[^ ^12^, ^23^ ^]^ the cell suspension was prepared with (shaken) and without shaking (static) as control samples. The results of the trypan blue exclusion assay showed that the viability of the cells in the floating droplets was over 80% of that of control cells, indicating that the plasma membrane was not damaged (Figure 3b). In the proliferation assay, no significant difference in cell growth between static, shaken, and levitated cells was observed within 4 h of levitation (Figure 3c). Therefore, there was no obvious damage to the pDNA and cells in the levitated droplet during the transfection period, and the cells maintained their growth potential.

### 2.3 Effective Transfection Using Ultrasonic Levitation

**Figure 4:**
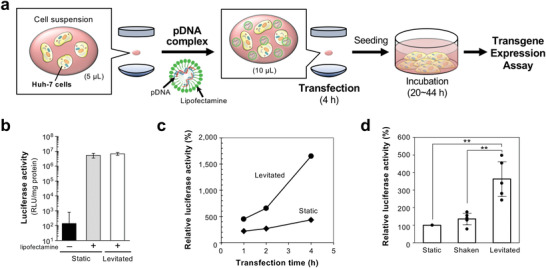
Luciferase transfection by ultrasonic levitation. a) Experimental scheme of cell transfection in a floating droplet generated by ultrasonic levitation. The cell suspension (5 µL) was ultrasonically levitated and a 5 µL pDNA complex solution was then added to the droplet. The mixed droplet was levitated for 4 h for transfection by adding sterile water every 15 min to avoid water evaporation. The transfected cells were seeded in a cell culture plate and incubated for 20–44 h, depending on the transfected transgene. b) Luciferase transfection. The transfection time of pDNA complex with cells in tube (static) or floating droplet (levitated) was 1 min, and the transfected cells were seeded in a cell culture plate. Data are presented as mean ± standard deviation (*n* = 3). c) Transgene expression efficiency depending on the transfection time of pDNA complex with cells. The transfection time of pDNA complex with cells was 1, 2, and 4 h, and the transfected cells were seeded in a cell culture plate. The transgene expression activity of the levitated culture (1 min) was normalized to 100%. d) Transgene expression efficiency determined by the luciferase assay. Luciferase activity of static, shaken, and levitated cells for 4 h transfection was measured. The transgene expression activity of the static culture in the tube was normalized to 100%. Data are presented as mean ± standard deviation (*n* = 5). Statistical analysis was performed using ANOVA followed by the Tukey–Kramer post‐hoc test. **p = 0.01.

**Figure** **4**a shows a typical flow diagram of the cell transfection in a floating droplet applied in the present study. Huh‐7 cells (8  × 10^4^ cells) were mixed with a complex of pDNA and a transfection reagent (lipofectamine) in a floating droplet generated using ultrasonic levitation. The transgene expression using ultrasonic levitation was evaluated using luciferase and EGFP reporter vectors. A pDNA–lipofectamine complex was transfected into Huh‐7 cells for 1 min in a floating droplet of culture medium (levitated) or in a tube without shaking (static). The transfected cells were then seeded into a cell culture plate and incubated at 37°C in 5% CO_2_ for 20 h. The luciferase activity in cells in the levitated culture (1 min levitation) corresponded to that in cells in the static culture (Figure 4b).

When the transfection time extended for 1 h or more, the luciferase activity in the levitated culture increased depending on the transfection time, whereas the activity in the static culture did not (Figure 4c). The luciferase activity in the levitated culture was confirmed to be dependent on the concentration of the complex solution (Figure S1a). In addition, the way to collect Huh‐7 cells from culture dish was investigated, and no difference between trypsinization and EDTA addition was observed (Figure S1b). From these results, we determined a requirement for effective transfection condition; the cells were harvested by trypsinization, and transfection of the pDNA–lipofectamine complex into Huh‐7 cells was performed for 4 h. Finally, the luciferase activity in cells in the levitated culture was significantly higher than that in cells in the static and shaken (in a tube) cultures (Figure 4d).

**Figure 5:**
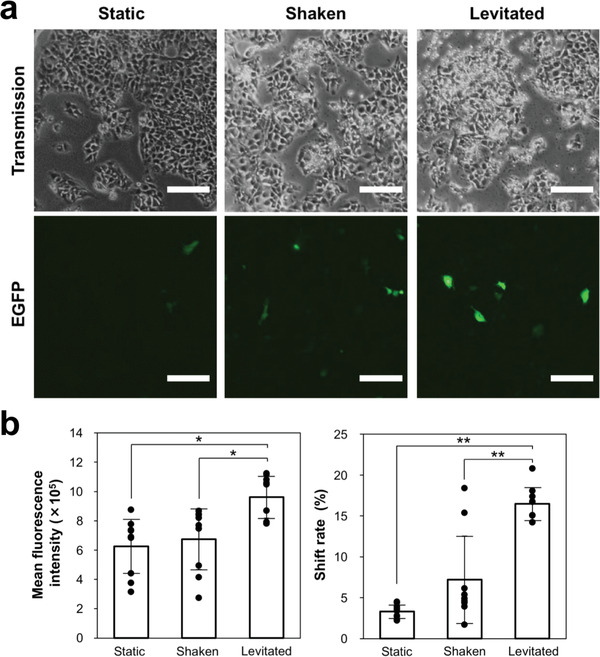
EGFP transfection by ultrasonic levitation. a) Optical microscopic images (upper) and EGFP fluorescent images in Huh‐7 cells (lower). b) Mean fluorescence intensity (left) and shift rate (right) of EGFP‐transfected cells were determined by flow cytometric analysis. Data are presented as mean ± standard deviation (*n* = 9). Statistical analysis was performed using the ANOVA followed by the Tukey–Kramer post‐hoc test (*p = 0.05, **p = 0.01).

The transgene expression efficiency using ultrasonic levitation was further evaluated using EGFP vector. A pDNA–lipofectamine complex was transfected into Huh‐7 cells for 4 h. EGFP expression in levitated cultures was increased compared to that in static and shaken cultures (**Figure** **5**a). Flow cytometry analysis showed that the mean fluorescence intensity of EGFP vector‐transfected cells in the levitated culture (9.6  × 10^5^) was approximately 1.5 times higher than that in the static (6.3  × 10^5^) and shaken (6.7  × 10^5^) cultures, and the shift rate of the levitated cells (16%) was also higher than that of the other cultures (3–7%) (Figure 5b). These results indicated that the expression level in the cells and the rate of EGFP‐expressing cells were increased by levitation.

### 2.4 Cellular Uptake and Its Pathway Impact the Transfection Efficiency

**Figure 6:**
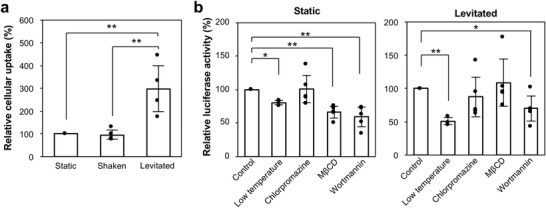
Changes in pDNA cellular uptake induced by levitation. a) Real‐time PCR showing an increase in the amount of pDNA in levitated cells. The amount of pDNA used in the static cell culture in the tube was normalized to 100%. Data are presented as mean ± standard deviation (*n* = 4). Statistical analysis was performed using the ANOVA followed by the Tukey–Kramer post‐hoc test. **p = 0.01. b) The effects of endocytic inhibitors on the transgene expression efficiency in static (left) and levitated (right) cell cultures. Huh‐7 cells were either precultured under cold conditions (low temperature, 7 °C) or pretreated with endocytic inhibitors (chlorpromazine, M*β*CD, and wortmannin). Subsequently, cells were transfected with the pDNA complex in a tube or levitated droplet. The transgene expression efficiency in control cells without inhibitors was normalized to 100%. Data are presented as mean ± standard deviation (*n* = 3 for low‐temperature conditions in the static and the levitated cultures, or n = 5 for the other conditions). Statistical analyses were performed using Welch's *t*‐test (*p = 0.05, **p = 0.01).

To elucidate the mechanism by which levitation induces higher transgene expression levels, the amount of pDNA uptake and its pathway were investigated in cells. The amount of pDNA in transfected cells was determined using real‐time PCR. The transfected cells were collected, and the extracted pDNA was subjected to PCR for quantitative analysis. The amount of pDNA uptake in cells in the levitated culture was three times higher than that in cells in the static culture (299 ± 101%; **Figure** **6**a).

**Figure 7:**
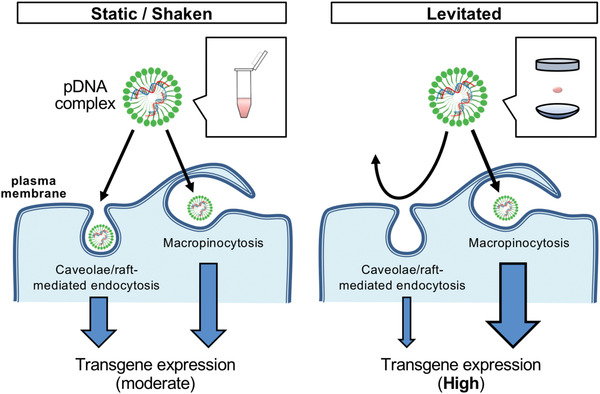
Efficient transfection in the levitated droplet by the consolidation of the endocytic uptake pathway into macropinocytosis.

To further clarify the cellular uptake pathways during levitation, the luciferase activity of cells pretreated at a low temperature ^[^ ^24^, ^25^ ^]^ or with endocytosis inhibitors^[^ ^26^, ^27^, ^28^ ^]^ was examined. The transgene expression level in the static and levitated cultures significantly decreased after pretreatment at a low temperature (7°C), indicating that the transfection was mediated by endocytosis (Figure 6b). As for the static culture, pretreatment with the endocytic inhibitors, methyl‐*β*‐cyclodextrin (M*β*CD) and wortmannin, significantly inhibited cellular uptake, suggesting that caveolae‐mediated endocytosis and macropinocytosis are major pathways for cellular uptake for subsequent transgene expression of the pDNA complex (Figure 6b, *left*). In contrast, the transgene expression level in cells in the levitated culture only decreased after pretreatment with wortmannin (Figure 6b, *right*). In view of the increase of the amount of pDNA uptaken into cells, this result demonstrates the inhibition of caveolae‐mediated endocytosis and suggests that the multiple endocytic uptake pathways were consolidated into macropinocytosis via levitation (**Figure** **7**). Our results demonstrate that ultrasonic levitation has the potential to be used as an effective culture condition by altering the endocytosis pathway.

## 3 Discussion

### 3.1 Efficient Transfection Mechanism

Contactless cell transfection in a levitated droplet was developed using planar transport and multiple droplet processing by the Foresti group.^[^ ^17^, ^18^ ^]^ Droplet mixing of the cell suspension and pDNA complex droplets was completed within a few minutes, and these cells were seeded into a 96‐well plate as per conventional procedures. In this study, we aimed to achieve cell growth, transfection, and gene expression in floating droplets. Therefore, we performed levitation transfection and gene expression and found that our method resulted in a highly efficient transfection by extending the transfection time. Efficient transfection is speculated to be achieved by improving the cellular uptake of the pDNA complex into the target cells by the internal flow of the ultrasonically levitated droplet.^[^ ^12^, ^23^ ^]^ Attaching the pDNA complex to the cell surface is required for the first step of intracellular uptake, and convective transport of cells and pDNA complex by the internal flow in the droplet might provide efficient intracellular uptake. However, this hypothesis was not supported by our results of cells in the shaking culture, which showed moderate transgene expression. Therefore, we hypothesized that unexpected interactions around the membrane microenvironment may occur after the attachment of the pDNA complex to the cell surface. In this study, the uptake of the pDNA complex with the transfection reagent, lipofectamine, occurred through two endocytic pathways: caveolae‐mediated endocytosis and macropinocytosis (Figure 7). Unexpectedly, the endocytic inhibitor treatment showed that the endocytic uptake pathways were consolidated into macropinocytosis under ultrasonic levitation. Therefore, we concluded that ultrasonic levitation affected the cell characteristics during transfection. Macropinocytosis vesicles typically form large vesicles called macropinosomes (>1 µm diameter), where micropinocytosis is induced by actin assembly.^[^ ^29^ ^]^ In contrast, the endocytic vesicle of caveolae‐mediated endocytosis is induced with caveolae coat proteins, such as caveolin‐1, and its size is smaller than that of macropinosome (∼50–60 nm in diameter). Thus, we conclude that the consolidation of the endocytic pathways into macropinocytosis resulted in a large endocytic vesicle, which resulted in an increase in pDNA uptake by cells and a high transgene expression level.

The endocytic pathways of macropinocytosis require actin; deforming the plasma membrane and bringing the material into the cytoplasm are involved in the polymerization of actin filaments or the action of myosin motor proteins on actin filaments.^[^ ^30^ ^]^ Fujimoto et al. demonstrated that endocytosis in A431 cells assayed in a suspension was inhibited by chemical compounds, which perturb intracellular actin dynamics, whereas neither compound inhibited endocytosis in adherent A431 cells.^[^ ^31^ ^]^ Furthermore, cell migration‐related actin bundles are affected by propagating acoustic waves on culture substrates.^[^ ^32^ ^]^ These studies supports our results, but details of actin dynamics of cells in the levitated droplet have not been further investigated.

### 3.2 The Limitations of An Extended Levitation Time

In the present study, the levitation transfection time was extended from a few minutes to four hours. In general, transfection time is the period of interaction between cells and the pDNA complex; therefore, it generally requires several hours. However, we observed that as the levitation time increased, the volume of water evaporated from the droplet increased. In addition, long‐term exposure to ultrasonic waves may damage DNA and cells. Therefore, we developed a droplet‐monitoring system and stringently investigated the damage to DNA and cells.

First, we resolved the limitation of water evaporation from the droplet by manually adding water every 5–10 min during transfection. The volume of the levitated droplet on the nanoliter scale was calculated in real‐time using a combination of a high‐speed camera and dedicated software, and the accurate volume of the culture medium was monitored during levitation.

The second issue is the possible damage to pDNA and cells during transfection by ultrasonic levitation. In general, biological damage caused by ultrasonic exposure is induced by cavitation, which generates reactive oxygen species and physical stress.^[^ ^20^ ^]^ In our study, we did not observe a remarkable degradation of pDNA in the ultrasonically levitated droplet for up to four hours. Additionally, damage to genomic DNA induced by ultrasonic exposure would result in a remarkable change in cell proliferation. However, we did not observe a significant change in cell proliferation among the levitated, static, and shaken cells. Moreover, most cells (over 80%) were not stained by trypan blue, indicating that the plasma membrane was not remarkably disrupted during four hours of levitation. We speculate that the damage to the plasma membrane observed in less than 20% of the cells in the levitated droplet is caused by mechanical/physical stress; therefore, using cell protectants, such as poloxamer and polyvinyl alcohol,^[^ ^33^, ^34^ ^]^ may enhance cell viability.

## 4 Conclusion

In this study, we developed a cell transfection method for floating droplets using ultrasonic levitation. The transgene expression efficiency and cellular uptake in the levitated droplets were significantly higher than those in traditional tubes. Surprisingly, the findings suggest that the endocytic uptake pathway was consolidated into macropinocytosis, indicating that ultrasonic levitation may alter cell characteristics. This study suggests that by manipulating the conditions for cell transfection in the levitated droplet, external proteins can be expressed in the cells without inducing DNA damage. In the present study, cell‐containing floating droplets were simply levitated for cell transfection and biochemically investigated. As recent technologies using oscillation, subwavelength, and multiple axis levitators have enabled dynamic manipulation of the levitated droplet,^[^ ^17^, ^35^, ^36^ ^]^ drastic enhancement of transfection would be realized in the future. Together with these improvements, the contactless and levitating transfection method can be widely applied in the field of cell engineering and has the potential to achieve plastic‐free, containerless processing.

## 5 Experimental Section

### Single‐Axis Ultrasonic Levitator with a High‐Speed Camera

A single‐axis acoustic levitator composed of a Langevin‐type ultrasonic transducer device with a frequency of 60 kHz (Honda Electronics Co., Ltd., Aichi, Japan) was used as previously described.^[^ ^8^ ^]^ The acoustic forces generated by the standing sound waves were manually adjusted by controlling the distance (10–15 mm) between the reflector and the bottom of the horn. The volume of the levitated droplet was measured with a custom‐ordered measurement system using a high‐speed camera with a complementary metal‐oxide‐semiconductor image sensor (Icomes Lab., Co., Ltd., Morioka, Japan). The droplet image was captured using a light‐emitting diode (LED) light source as a backlight, and its volume was then calculated automatically in real‐time. The system was located in a laboratory at 22 °C or a cold room at 7 °C, and the temperature and humidity were monitored using a data logger (Thermo Recorder TR72wb, T&D Corporation, Matsumoto, Japan). The temperature of the levitated droplet was measured using a thermal camera (FLIR C2; FLIR Systems AB, Taby, Sweden).

### Preparation of pDNA Expression Vector

An EGFP‐N1 vector (Clontech Laboratories, Mountain View, CA, USA) encoding the enhanced green fluorescent protein gene and a pGL4.51 vector (Promega Corporation, Madison, WI, US) encoding the luciferase gene were amplified in *E. coli* (DH5*α*) cells and then purified using an endotoxin‐free Plasmid Giga Kit (QIAGEN, Hilden, Germany) according to the manufacturer's instructions. The pDNA stock solution was adjusted to 1 mg mL^−1^ with UltraPure DNase/RNase‐Free distilled water (Thermo Fisher Scientific, Waltham, MA, USA).

### Cell Culture

Human hepatocellular carcinoma Huh‐7 cells were purchased from the Riken Cell Bank (Ibaraki, Japan) and maintained in Dulbecco's modified Eagle's medium (DMEM) containing 10% fetal bovine serum (FBS), 100 U/mL penicillin, and 100 µg/mL streptomycin at 37°C in a 5% CO_2_ humidified atmosphere.^[^ ^37^ ^]^ For the levitation experiments, Huh‐7 cells were harvested by trypsinization (or 0.5 mM EDTA at pH 7.4 in PBS) and then suspended in DMEM containing 10% FBS at a concentration of 8  × 10^3^ cells/µL (Huh‐7 cell suspension).

### Preparation of the pDNA–lipofectamine Complex

Six microliters of lipofectamine 2000 transfection reagent (Invitrogen, Carlsbad, CA, USA) were diluted with 94 µL Opti‐MEM I Reduced Serum Medium (Gibco, Waltham, MA, USA). One hundred microliters of the diluted lipofectamine and 100 µL of the pDNA solution in Opti‐MEM I Reduced Serum Medium (60 µg/mL) were gently mixed, and the mixture was then incubated for 15 min at 22–25°C to obtain a pDNA/Lipofectamine complex. The final concentration of the pDNA in the solution was 3–150 µg/mL.

### pDNA Stability During Ultrasonic Levitation

The pDNA stock solution was diluted to 30 µg/mL with phosphate‐buffered saline (PBS). The diluted pDNA solution was subjected to ultrasonic levitation for 0, 0.5, 1, and 4 h. After levitation, the diluted pDNA solutions were collected in a 0.5 mL tube, and the pDNA concentration was measured using NanoDrop Lite (Thermo Fisher Scientific). pDNA (150 ng per well) was electrophoresed on a 1.0% agarose gel in Tris‐acetate‐EDTA (TAE) buffer for 30 min at 100 V and stained with ethidium bromide.

### Trypan Blue Exclusion Assay

Ten microliters of Huh‐7 cell suspension (8  × 10^3^ cells/µL) were ultrasonically levitated for 1 and 4 h. After levitation, the collected cell suspension was diluted with 400 µL DMEM containing 10% FBS in a 0.5 mL tube. Fifty microliters of the cell suspension were mixed with an equal amount of 0.4% trypan blue solution in PBS. Blue (dead) and white (living) cells were counted using a hemocytometer and observed under an inverted microscope.

### Cell Proliferation Assay

Ten microliters of Huh‐7 cell suspension (8  × 10^3^ cells/µL) were ultrasonically levitated for 1 and 4 h. After levitation, the collected cell suspension was diluted with 400 µL of DMEM containing 10% FBS in a 0.5 mL tube. Cells were seeded in 96‐well plates at a density of 3000 cells/well in DMEM containing 10% FBS and cultured for 24 h for cell attachment (the initial time point). Cells were additionally cultured for 24 or 48 h to evaluate their proliferation ability. Thereafter, 5 mM water‐soluble tetrazolium salt (WST)‐1 (Dojindo Laboratories, Kumamoto, Japan) and 0.2 mM 1‐methoxy‐5‐methylphenazinium methyl sulfate (1‐methoxy PMS) solution in PBS were added to the 96‐well plates. After 4 h, the absorbance was measured at 450 nm, with a reference wavelength of 690 nm, using a microplate reader (PowerScan HT, DS Pharma Biomedical, Osaka, Japan). The results were calculated as the percentage of control cells at the initial time point.

### Cell Transfection Using a Floating Droplet (Levitating Transfection)

Five microliters of the Huh‐7 cell suspension (8  × 10^3^ cells/µL) were levitated ultrasonically, and 5 µL of the pDNA–lipofectamine complex solution (3, 30, and 150 µg/mL) was added to the suspension. During transfection of the levitated droplet at 22°C, the shape of the droplet was captured with a high‐speed camera to monitor the droplet volume, and sterile water (3–4 µL) was added every 5–10 min to avoid volume reduction. After 4 h of ultrasonic levitation, transfected cells were seeded in 24‐well plates, followed by incubation at 37°C in a 5% CO_2_ humidified atmosphere for 20 h for the luciferase assay or 44 h for the EGFP expression assay.^[^ ^37^ ^]^

### Endocytosis Inhibition

The effect of endocytosis inhibition was evaluated by treating cells at a low temperature. After pre‐incubation of the cells in a 1.5 mL tube at 7°C for 1 h, cell transfection in the floating droplet was conducted for 4 h at 7°C.

Cells were pretreated with the endocytic inhibitors wortmannin (150 µM) for 60 min, methyl‐*β*‐cyclodextrin (M*β*CD) (1.5 mM) for 60 min, and chlorpromazine (30 µg/mL) for 30 min at 37°C in a 5% CO_2_ humidified atmosphere. Subsequently, the cells were transfected with the pDNA complex in the floating droplet, and transgene expression efficiency was determined using a luciferase assay.

### Luciferase Assay

After transfection, the cells were washed thrice with PBS and lysed with a cell lysis reagent. Luciferase gene expression was evaluated using a luciferase assay system (code E1500, Promega). Light unit values were measured using a luminometer (Gene Light GL‐220, Microtec Co. Ltd., Chiba, Japan) and normalized to the total protein content of the cell lysate. Protein concentrations were determined using a DC Protein Assay Kit (Bio‐Rad Laboratories, Hercules, CA, USA).

### EGFP Expression Assay (Flow Cytometry)

Transmission and fluorescence cell images were acquired using an inverted fluorescence microscope (IX‐71; Olympus). After microscopic observation, the cells were washed thrice with ice‐cold PBS, harvested by trypsinization, and resuspended in 0.5% bovine serum albumin (BSA)/PBS. The mean fluorescence intensity was recorded at 520 nm after excitation at 488 nm using a flow cytometer (BD Accuri C6; BD Biosciences, Franklin Lakes, NJ, USA). A total of 10000 individual cells were analyzed for each sample.

### Isolation of DNA from Transfected Cells and Real‐time Polymerase Chain Reaction (RT‐PCR)

After 4 h of ultrasonic levitation, the pDNA complex associated with the cell surfaces was removed using CellScrub Buffer (Gene Therapy Systems, Inc., San Diego, CA, USA). Transfected cells were pelleted by centrifugation at 900  × g for 5 min and resuspended in a buffer. After incubation for 15 min at 22–25°C, the cells were pelleted by centrifugation at 900  × g for 5 min. The cells were resuspended in PBS and the suspension was centrifuged at 900  × g for 5 min. Cells were washed thrice with PBS. Total DNA was isolated from transfected cells using a DNeasy Blood & Tissue Kit (QIAGEN) according to the manufacturer's instructions.

RT‐PCR was performed in 10 µL of SYBR Premix Ex Taq II (Takara Bio, Shiga, Japan) and total DNA using the PikoReal 96 Real‐Time PCR System (Thermo Fisher Scientific). The levels of glyceraldehyde‐3‐phosphate dehydrogenase (GAPDH) transcripts were used to calculate the number of cells and normalize DNA levels. Primer sequences for luciferase and GAPDH were designed as follows: forward luciferasene, 5′‐CAGCAAGGAGGTAGGTGAGG‐3′; reverse luciferasene, 5′‐TCTTACCGGTGTCCAAGTCC‐3′; forward GAPDH, 5′‐ATGGGGAAGGTGAAGGTCG‐3′; reverse GAPDH, 5′‐TAAAAGCAGCCCTGGTGACC‐3′.

### Statistical Analysis

All experiments were repeated in triplicate, unless specified. Data are presented as mean ± standard deviation. The significance was determined using the one‐way analysis of variance (ANOVA) followed by the Tukey–Kramer post‐hoc test (or Welch's *t*‐test). Results with probability (*p*) values less than 0.05 (*) and 0.01 (**) were considered significant and highly significant, respectively, and results with *p* values greater than 0.05 were considered insignificant. Data were analyzed using Microsoft Excel software.

## Conflict of Interest

The authors declare no conflict of interest.

## Supporting information

Supporting Information

## Data Availability

The data that support the findings of this study are available on request from the corresponding author. The data are not publicly available due to privacy or ethical restrictions.
