# Supplementary material for: Effective Cell Transfection in An Ultrasonically Levitated Droplet for Sustainable Technology
Source: Adv Sci (Weinh). 2022 Aug 26;9(30):2203576. doi: 10.1002/advs.202203576 (PMC9596829; doi:10.1002/advs.202203576)
Supplement: Supplementary file 1 — Supporting Information [file ADVS-9-2203576-s001.pdf]

Supporting Information

Effective cell transfection in an ultrasonically levitated droplet for sustainable technology

*Takahiro Arai, Toshinori Sato, and Teruhiko Matsubara**

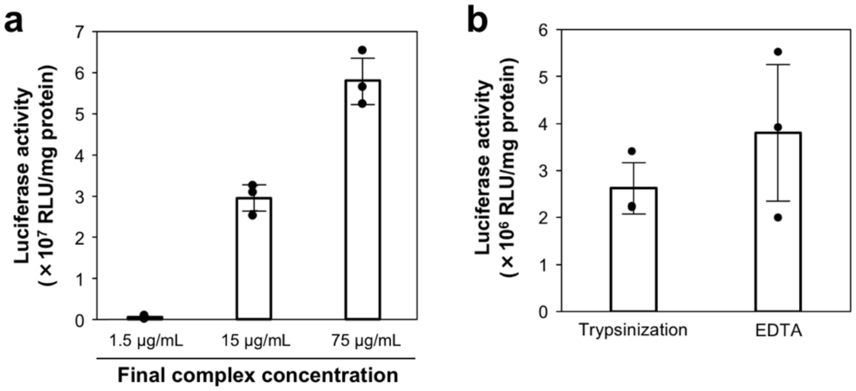

Figure S1. Determination of detailed condition for luciferase transfection by ultrasonic levitation.

(a) Luciferase activity depending on the concentration of pDNA complex. The pDNA–lipofectamine complex solution (3, 30, and 150 μg/mL) was added to the levitated cell suspension.

(b) Luciferase activity depending on the preparation of Huh-7 cell suspension. Huh-7 cells were harvested by trypsinization or EDTA addition.

Transfection and post-transfection time, 4 h and 20 h, respectively.
